# Spontaneous retroperitoneal hematoma after COVID-19 infection: A case report

**DOI:** 10.1097/MD.0000000000041077

**Published:** 2025-01-10

**Authors:** Xi Chen, Zheng Li, Liangping Zou, Yupin Lan, Xiaoling Wu, Hui Wang

**Affiliations:** a Department of Gastroenterology, Zhangzhou Traditional Chinese Medicine Hospital, Zhangzhou, China; b Department of Gastroenterology, Mulei County People’s Hospital, Mulei, China; c Fujian University of Traditional Chinese Medicine, Fuzhou, China; d Chinese Medicine Hospital Changji Hui Autonomous Prefecture, Changji, China; e Jian’ou Municipal Hospital, Nanping, Fujian, China; f Zhangzhou Dermatological Prevention and Treatment Hospital, Zhangzhou, China; g College of Acupuncture-Moxibustion and Tuina, Beijing University of Chinese Medicine, Beijing, China.

**Keywords:** abdominal pain, anticoagulation, antiplatelet, COVID-19, shock, spontaneous retroperitoneal hematoma

## Abstract

**Rationale:**

Spontaneous retroperitoneal hematoma (SRH) is a rare but potentially fatal condition, often associated with anticoagulation therapy. With the global prevalence of COVID-19 and the widespread use of anticoagulants in its management, there is an increasing need to recognize rare but serious complications like SRH. This case report aims to emphasize the importance of early recognition and intervention of SRH in patients with COVID-19 undergoing anticoagulation therapy, to improve patient outcomes and reduce mortality.

**Diagnoses:**

An 86-year-old male with a history of COVID-19 presented with recurrent cough, hemoptysis, and fever. Initial treatment included antiviral and anticoagulant therapy. The patient later developed abdominal distension, pain, and eventually hypovolemic shock, leading to the diagnosis of SRH confirmed by imaging and a significant drop in hemoglobin levels.

**Interventions:**

The patient received comprehensive supportive care, including noninvasive ventilation, antiviral therapy, and anticoagulants. Upon the onset of SRH, emergency interventions included fluid resuscitation, vasopressors, and interventional embolization of the bleeding vessels.

**Outcomes:**

The patient initially responded well to COVID-19 treatment but developed SRH, which was managed successfully with interventional embolization. Post-procedure, the patient’s vital signs stabilized, hemoglobin levels gradually increased without the need for further transfusions, and he regained full consciousness. Over the following weeks, the patient showed continuous improvement, with resolution of abdominal pain and a return to baseline mobility. He was discharged in stable condition with scheduled follow-ups.

**Lessons:**

This case underscores the critical need for vigilance in monitoring patients on anticoagulation therapy, particularly those with COVID-19, for signs of SRH. Early recognition and prompt intervention are essential to improve outcomes. Clinicians should maintain a high index of suspicion for SRH in patients presenting with unexplained abdominal pain and hypovolemic shock, even in the absence of typical risk factors.

## 1. Introduction

Spontaneous retroperitoneal hematoma (SRH) is a rare condition that was first reported in 1909. Its pathogenesis is complex and can be caused by various factors. Clinical manifestations are not typical and may be easily missed in the early stages. As the disease progresses, symptoms may appear and be misdiagnosed. If blood loss continues to increase, more severe symptoms may occur. SRH can occur in any part of the blood vessels in the abdominal cavity. Treatment depends on the size of the ruptured vessel and may require surgical intervention.

## 2. Case report

**Figure 1.**
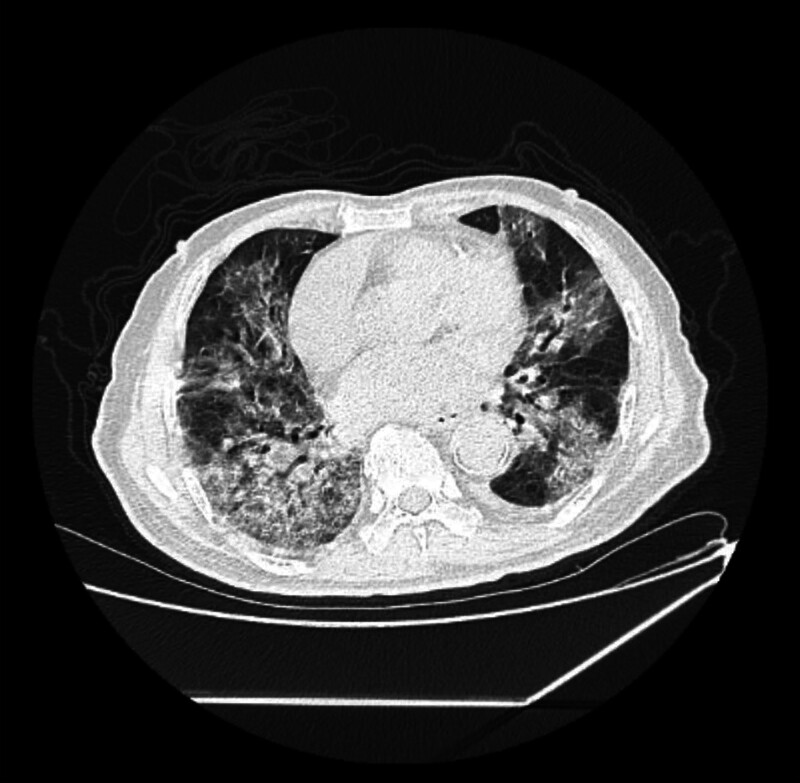
Chest CT image before treatment.

An 86-year-old male patient presented with a 3-day history of recurrent cough, hemoptysis, and fever. Upon examination, his body temperature was recorded at 38.1°C and he reported experiencing palpitations, chest tightness, and dyspnea. Auscultation revealed coarse respiratory sounds in both lungs and the presence of wet rales in the lower lungs bilaterally. No other significant findings were noted. A chest CT scan indicated the presence of viral pneumonia and multiple gallstones with cholecystitis (Fig. 1). Laboratory tests showed elevated markers of inflammation and a hypercoagulable state. The patient tested positive for COVID-19 antibodies.

**Figure 2.**
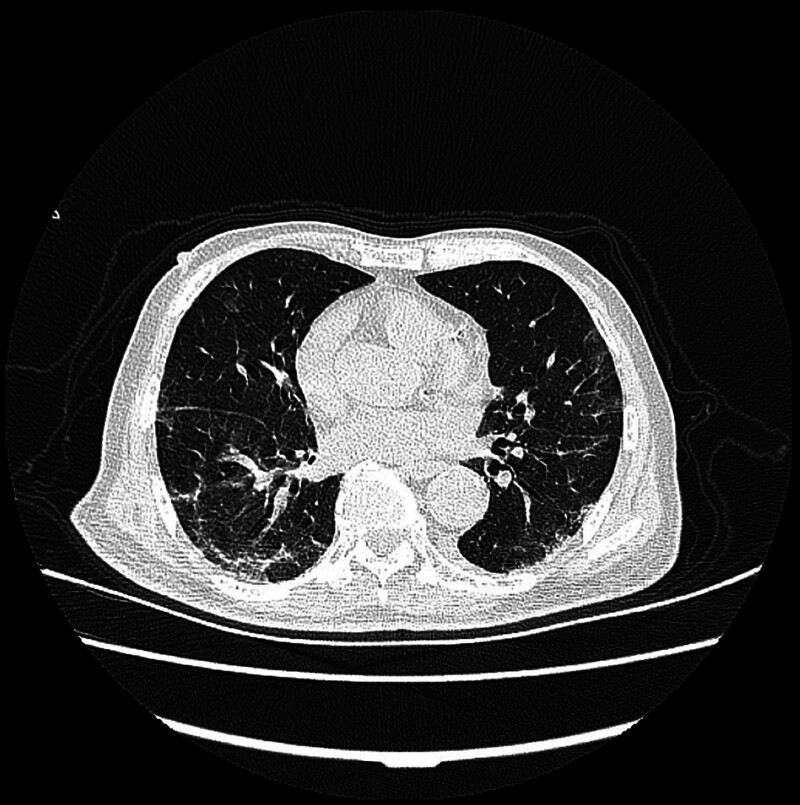
Chest CT image after treatment.

Upon admission, the patient was treated with noninvasive ventilation, antiviral therapy, antibiotics, anti-inflammatory agents, anticoagulants, expectorants, cough suppressants, acid inhibitors, gastric mucosal protectants, electrolyte replacement, and nutritional support. His symptoms gradually improved and a follow-up CT scan showed a reduction in inflammation (Fig. 2). After 1 week of treatment, the patient was able to tolerate intermittent cessation of ventilatory support, and after 10 days, the ventilator was discontinued.

Subsequently, the patient developed abdominal distension and soreness as well as weakness in his waist and legs. Bed rest was considered as a possible cause, and he was treated with a plaster for pain relief. The soreness and weakness improved the following day, but abdominal distension and mild abdominal pain persisted. Later that day, the patient experienced worsening abdominal pain and suddenly lost consciousness. He was unresponsive to stimuli and had weak breathing with a pulse rate of 120 to 130 beats/min and blood pressure of 60 to 70/30 to 40 mm Hg. Both pupils were 3 mm in diameter with a dull response to light. Breath sounds were low in both lungs, and heart sounds were low and dull. The neurology department and ICU were urgently consulted for rescue treatment.

After receiving treatment including vasopressors, fluid resuscitation, and vasodilators, the patient regained consciousness. Physical examination revealed a pulse rate of 120 to 130 beats/min, blood pressure of 110 to 130/70 to 80 mm Hg, bilateral pupils of 3 mm that were responsive to light, coarse breath sounds in both lungs, low and dull heart sounds, tension in the left abdominal wall, and bowel sounds at a rate of 2 to 3 times/min. A bedside color Doppler ultrasound showed evidence of intra-abdominal bleeding.

**Figure 3.**
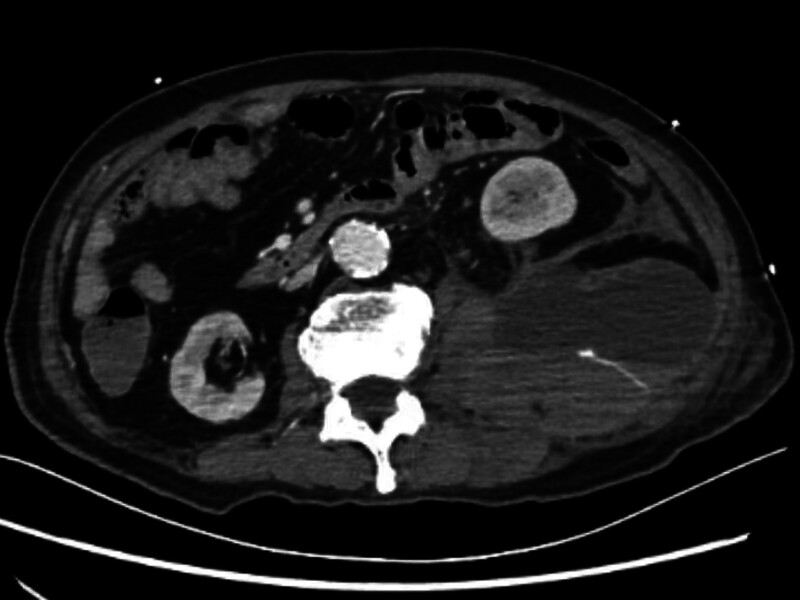
Emergency abdominal CT with contrast showed retroperitoneal hemorrhage.

**Figure 4.**
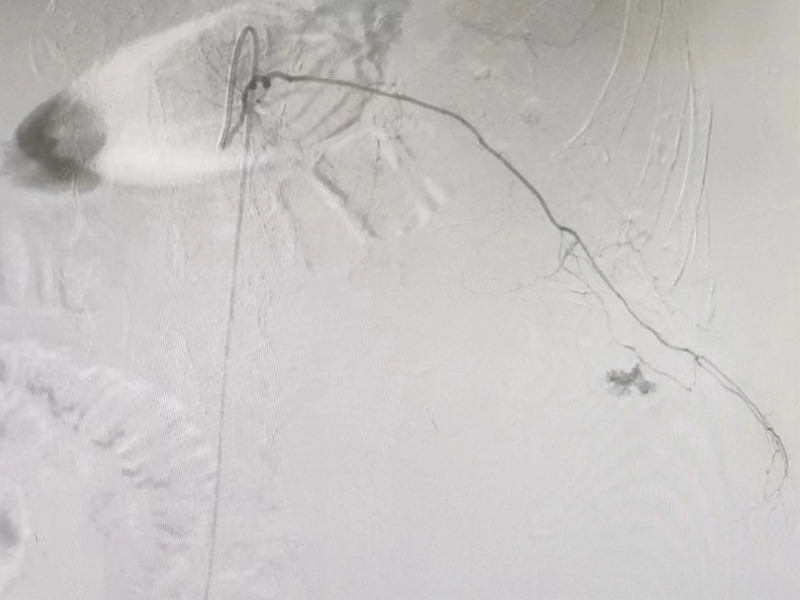
Contrast agent extravasation was observed in the left 12th intercostal artery.

**Figure 5.**
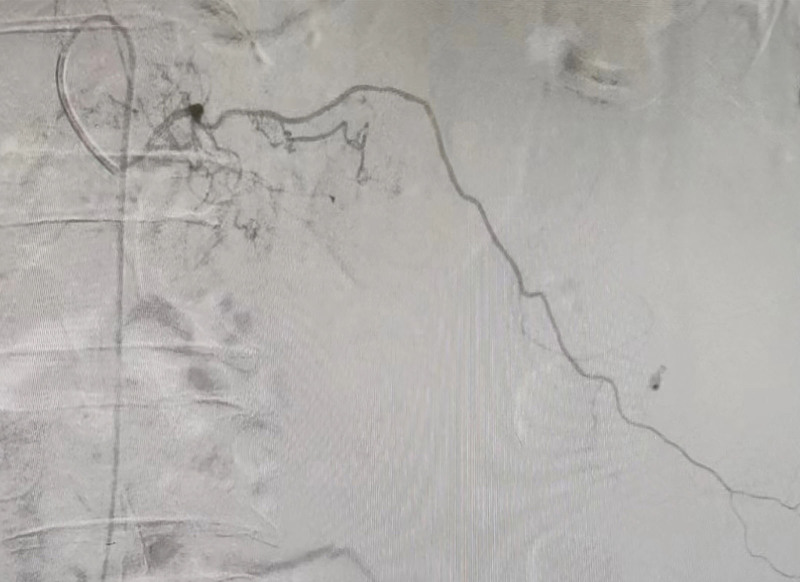
Contrast agent extravasation was observed in the left lumbar artery.

An urgent contrast-enhanced whole-abdomen CT scan (Fig. 3) showed SRH, and an emergency blood test revealed a decrease in hemoglobin from 123 to 74 g/L (reference range: 110–170 g/L). As the patient’s vital signs were relatively stable, interventional surgery was performed. During the procedure, contrast extravasation was observed in the left T12 intercostal artery and left lumbar artery (Figs. 4 and 5), and successful vascular embolization was performed.

## 3. Outcomes

Post-embolization, the patient’s hemodynamic status improved significantly. His blood pressure stabilized within normal ranges, and his heart rate decreased to 80 to 90 beats/min. Hemoglobin levels were monitored daily and showed a gradual increase without the need for additional blood transfusions. The abdominal distension and pain subsided over the next few days. The patient was mobilized gradually, and his respiratory function continued to improve without the need for ventilatory support. He was discharged after 3 weeks of hospitalization with instructions for follow-up care and monitoring.

## 4. Discussion

SRH is a rare and potentially fatal disease with a high misdiagnosis rate. According to statistics, the 1-month fatality rate of patients with SRH is 10.1%.^[1]^ The most typical symptoms are abdominal pain, ecchymosis, and hypovolemic shock, along with back and leg pain, difficulty breathing, and fainting. However, only one-third of patients will show hemodynamic instability such as hypotension and shock,^[2]^ which is often overlooked clinically. In this case, the patient developed abdominal distension and back and leg soreness 10 days after treatment. Initially, these symptoms were attributed to the incomplete recovery of gastrointestinal function after resuming oral intake and prolonged bed rest in the elderly. The symptoms improved after symptomatic treatment such as pain relief, which seemed to confirm our hypothesis. However, these were actually signs of SRH. The symptom relief was probably due to the reduced hematoma compression after SRH bleeding, resulting in a false impression of improvement while the condition was actually worsening, leading to a sudden onset.

SRH is commonly attributed to the use of anticoagulants and antiplatelet drugs. However, anticoagulants themselves are not related to bleeding in up to one-third of patients. SRH should be suspected in patients with lower body pain, regardless of anticoagulant use, especially if they have signs of poor blood perfusion. Moreover, SRH should be considered for patients with acute hemorrhagic anemia of unknown origin.

As of the beginning of October 2022, 615 million cases of COVID-19 have been reported globally, with 6.5 million fatalities.^[3]^ Hospitalized patients with COVID-19 possess a higher risk for hypercoagulable state and thrombotic complications. The pathophysiology behind this has been suspected to result from increased pro-inflammatory cytokines.^[4]^ The reinfection rate of COVID-19 is 5% to 15%, but this proportion may increase over time.^[5]^ Analysis of data from approximately 1.6 million adults showed that >3-quarters of those reinfected had mild infections both times. Compared to patients with mild or moderate first infections, those with severe first infections had more severe cases (7.4%) and a higher mortality rate (5.7%) upon reinfection.^[6]^ Additionally, 30% of patients who used a ventilator during their first infection required hospitalization upon reinfection.^[1]^ Those who were severely ill during their first infection remain vulnerable upon reinfection.^[7]^ However, due to its strong infectivity, even a small rate of severe illness and fatality combined with a large population base would still result in a significant number of patients at risk. In this case, the patient had a severe case of novel coronavirus pneumonia and was bedridden and hypercoagulable due to his advanced age. He received routine anticoagulant treatment without contraindications, which increased the risk of SRH.

The retroperitoneal space can contain a large amount of blood. Therefore, patients with SRH who have hypoperfusion or acute hemorrhagic anemia should receive prompt interventions, such as shock management, hemodynamic stabilization, and discontinuation of anticoagulant/antiplatelet drugs. Early consultation is also essential. Surgery or interventional embolization can be effective, especially for patients with abnormal vascular bleeding. Statistics show that about 25% of patients require interventional treatment and 7% require surgery. In this case, the patient had a sudden loss of consciousness, tachycardia, and hypotension. Although the vital signs stabilized after resuscitation, the heart rate remained high, suggesting ongoing bleeding. Therefore, interventional treatment was performed.

Studies have shown that active anticoagulant therapy is recommended for severe patients, and anticoagulant therapy is recommended for those without contraindications. Anticoagulant and antiplatelet therapy should be continued after suffering from COVID-19 for patients with underlying diseases unless there is severe bleeding or other contraindications.^[8–12]^ However, in addition to saving lives, this also increases the risk of SRH, especially for some patients who improve their condition after treatment and complain of abdominal pain and bloating. Therefore, we should remain vigilant during this special period even if some patients are relieved after symptomatic treatment.

## 5. Limitations

This case report has several limitations. Being a single case study, the findings may not be generalizable to all patients with COVID-19 and SRH. Due to the emergency nature of the patient’s condition, comprehensive diagnostic evaluations were limited. Additionally, long-term follow-up was not possible, restricting the understanding of prolonged outcomes. Further research with larger sample sizes is necessary to better understand the association between COVID-19, anticoagulation therapy, and the risk of SRH.

## Author contributions

**Conceptualization:** Xi Chen, Liangping Zou, Yupin Lan, Xiaoling Wu.

**Data curation:** Liangping Zou, Yupin Lan.

**Formal analysis:** Liangping Zou, Hui Wang.

**Funding acquisition:** Yupin Lan.

**Investigation:** Xi Chen, Zheng Li, Liangping Zou, Yupin Lan, Xiaoling Wu, Hui Wang.

**Methodology:** Zheng Li, Xiaoling Wu, Hui Wang.

**Project administration:** Zheng Li.

**Resources:** Zheng Li.

**Supervision:** Xiaoling Wu.

**Validation:** Xi Chen, Xiaoling Wu.

**Writing – original draft:** Xi Chen.

**Writing – review & editing:** Hui Wang.
